# Phenotypic Variation in Disease Severity Among Hospitalized Pediatric Patients With COVID-19: Assessing the Impact of COVID-19 in the EPICO Study

**DOI:** 10.3389/ijph.2025.1607246

**Published:** 2025-03-18

**Authors:** María Camila Sossa-Alarcón, Mónica Paola Gutiérrez, Natalia Becerra, Luz Yessenia Ortegon, María Camila David, Melisa Naranjo Vanegas, Gabriela Friedrich, Pablo Vásquez-Hoyos, María Lucía Mesa-Rubio, Luis Miguel Navarro-Ramirez, Sergio Moreno-Lopez, Olga Lucía Baquero, Luz Marina Mejía, Juan Gabriel Piñeros, Sonia Restrepo-Gualteros, Carlos Álvarez-Moreno, Alejandro Díaz-Díaz, Iván Gutierrez-Tobar, Andrés Camilo Mesa, William Ricardo Bachiller Tuta, Clara Esperanza Galvis Diaz, Martha Africano, José Manuel Nieto, Paola Marsela Pérez Camacho, Claudia Beltrán-Arroyave, Rosalba Vivas Trochez, Irati Gastesi, Cinta Moraleda, Alfredo Tagarro García, Blanca Herrero, Lourdes Calleja, Carlos Grasa, Paula Rodriguez, Susana Melendo, Antoni Soriano-Arandes, Irene Gómez Pastrana, Sonsoles García García, Victoria Fumado, Andrea Ramírez Varela

**Affiliations:** ^1^ Pediatrics Department, Universidad de los Andes and Fundación Santa fe de Bogotá, Bogotá, Colombia; ^2^ School of Medicine, Universidad de los Andes, Bogotá, Colombia; ^3^ Medical Imagine & AI group - Bioscience Center, Ayudas Diagnósticas Sura, Medellín, Colombia; ^4^ Red Colaborativa Pediátrica de Latinoamérica (LaRed Network), Montevideo, Uruguay; ^5^ School of Medicine, Universidad Nacional de Colombia, Bogotá, Colombia; ^6^ Cancer and Molecular Medicine Research Group (CAMMO), Bogotá, Colombia; ^7^ Department of Pediatrics, Clínica Infantil Colsubsidio, Bogotá, Colombia; ^8^ Department of Pediatrics, Instituto de Ortopedia Infantil Roosevelt. Bogotá, Colombia; ^9^ Clínica Universitaria Colombia, Clínica Colsanitas Grupo Keralty, Bogotá, Colombia; ^10^ Department of Pediatrics, Hospital Pablo Tobón Uribe, Medellín, Colombia; ^11^ Department of Pediatrics, Hospital General de Medellín, Medellín, Colombia; ^12^ Red Neumocolombia, Bogotá, Colombia; ^13^ Clínica Infantil Santa María del Lago, Clínica Colsanitas Grupo Keralty, Bogotá, Colombia; ^14^ Department of Pediatrics, Hospital Infantil San José, Bogotá, Colombia; ^15^ Department of Pediatrics, Fundación Universitaria de Ciencias de la Salud, Bogotá, Colombia; ^16^ Department of Pediatrics, Sociedad de Cirugía de Bogotá - Hospital de San José, Bogotá, Colombia; ^17^ Department of Pediatrics, Hospital Militar Central, Bogotá, Colombia; ^18^ Department of Pediatrics, Clínica Materno Infantil San Luis, Bucaramanga, Colombia; ^19^ Department of Pediatrics, Universidad Industrial de Santander, Bucaramanga, Colombia; ^20^ Department of Pediatrics, Hospital Regional de la Orinoquía, Yopal, Colombia; ^21^ Pediatric Infectious Diseases Department, Fundación Valle del Lili, Cali, Colombia; ^22^ Faculty of Health Sciences, Universidad Icesi, Cali, Colombia; ^23^ Department of Pediatrics, Clínica del Rosario. Medellín, Colombia; ^24^ School of Medicine, Universidad de Antioquia, Medellín, Colombia; ^25^ Department of Pediatrics, Clínica SOMA, Medellín, Colombia; ^26^ Fundación Investigación Biomédica, Hospital Universitario 12 de Octubre (FIBH120), Instituto de Investigación, Hospital 12 de Octubre (imas12), Madrid, Spain; ^27^ Department of Pediatrics, Infanta Sofía University Hospital, European University of Madrid, Madrid, Spain; ^28^ Department of Pediatrics, Hospital Universitario Niño Jesús, Madrid, Spain; ^29^ Department of Pediatrics, Hospital Universitario La Paz, Madrid, Spain; ^30^ Department of Pediatrics, Hospital Universitario Vall D’Hebron, Barcelona, Spain; ^31^ Department of Pediatrics, Hospital Universitario Sant Joan de Deu, Barcelona, Spain; ^32^ Department of Epidemiology, School of Public Health, The University of Texas Health Science Center at Houston, Houston, TX, United States; ^33^ Department of Pediatrics, McGovern Medical School, University of Texas Health Sciences Center at Houston (UTHealth), Houston, TX, United States; ^34^ School of Public Health, Center for Health Equity, University of Texas Health Science Center at Houston, Houston, TX, United States

**Keywords:** COVID-19, pediatrics, inpatients, cluster analysis, SARS-CoV-2 variants

## Abstract

**Objective:**

To characterize the clinical phenotypes of SARS-CoV-2 infection in hospitalized children as part of the EPICO multicenter cohort study.

**Methods:**

We included hospitalized children with confirmed SARS-CoV-2 infection from Colombian and Spanish institutions to assess disease evolution and outcomes. Cluster analysis was performed to identify clinical phenotypes.

**Results:**

A total of 2318 patients were included (55% male, 36% infants). Five phenotype clusters emerged: Cluster 1 (26.5%): infants without comorbidities, low PICU admissions and mortality; Cluster 2 (18.5%): respiratory comorbidities, high microorganism co-detection and mortality; Cluster 3 (11.5%): fever, gastrointestinal symptoms, high PICU admissions; Cluster 4 (32%): mild unspecific symptoms, low mortality; Cluster 5 (11.3%): adolescents without comorbidities, low co-detection and hospitalization rates. Findings were consistent across both countries.

**Conclusion:**

Identifying clinical phenotypes of SARS-CoV-2 in children may improve risk stratification and guide future management strategies.

## Introduction

Since the onset of the COVID-19 (acronym for Coronavirus Disease of 2019) [1] pandemic in 2020, there has been extensive documentation of the disease in adult populations and diagnostic approaches that focus on symptom-based screening in children [2]. However, there is a lack of information and evidence regarding the manifestations, diagnosis, treatment, and prognosis of COVID-19 in the pediatric population, which may be significant for acute decision-making and prognosis.

The behavior of the virus in pediatric patients has been characterized by constant changes and unpredictability. In general, children with COVID-19 have a lower risk of hospitalization and severe complications [3]. Various studies have reported that up to 17% of pediatric patients are asymptomatic, while approximately 63% exhibit mild symptoms [4], emphasizing that the percentage of asymptomatic patients may be underestimated, as most do not get tested for SARS-CoV-2 [3]. Besides, we already know that COVID-19 is causing major physical, psychological, developmental, behavioral, and social health consequences for children, having an impact even in mortality [5, 6].

In 2020, a severe disease phenotype related to children infected with SARS-CoV-2 was initially identified in South London (UK) [7]. Following this discovery, a prospective cohort study conducted in England, Wales, and Scotland [8] described three distinct clusters of clinical phenotypes: discrete respiratory illness cluster, systemic mucocutaneous enteric illness cluster, and neurological illness cluster. Later another cross-sectional multicenter study, nested within the cohort EPICO-AE (Epidemiological Study of COVID-19 in Children of the Spanish Society of Pediatrics), also identified three phenotypes: Lower Respiratory, Gastrointestinal and Flu-like [2]. These investigations utilized symptom-based cluster analysis to categorize the disease presentations.

While some studies had focused exclusively on the multisystem inflammatory syndrome in children (MIS-C) and described phenotypes associated with the immune response to SARS-CoV-2 phenotype [9, 10], they have overlooked other disease presentations. Other studies have also differentiated between MIS-C and acute forms of the disease; however, they do not distinguish between the different acute presentations beyond a description of the symptoms that may occur [11, 12]. Similarly, other studies have described specific manifestations, such as neurologic or ophthalmological complications of the disease in pediatric patients, without including all possible presentations as distinct phenotypes [13, 14].

Furthermore, although certain studies, have mentioned clinical presentation, treatment and outcomes [2, 8, 11, 15] these aspects were not incorporated together into a cluster analysis, and, importantly, there is a lack of information regarding the population of Latin America, with only observational and descriptive data in terms of prevalence but a lack of data in terms of clinical presentation [16]. This study aims to describe and define the disease phenotypes among hospitalized pediatric population aged 29 days to 17 years, affected by SARS-CoV-2 infection included in the EPICO cohort in Colombia and Spain, using cluster analysis.

## Methods

### Study Design and Settings

**FIGURE 1:**
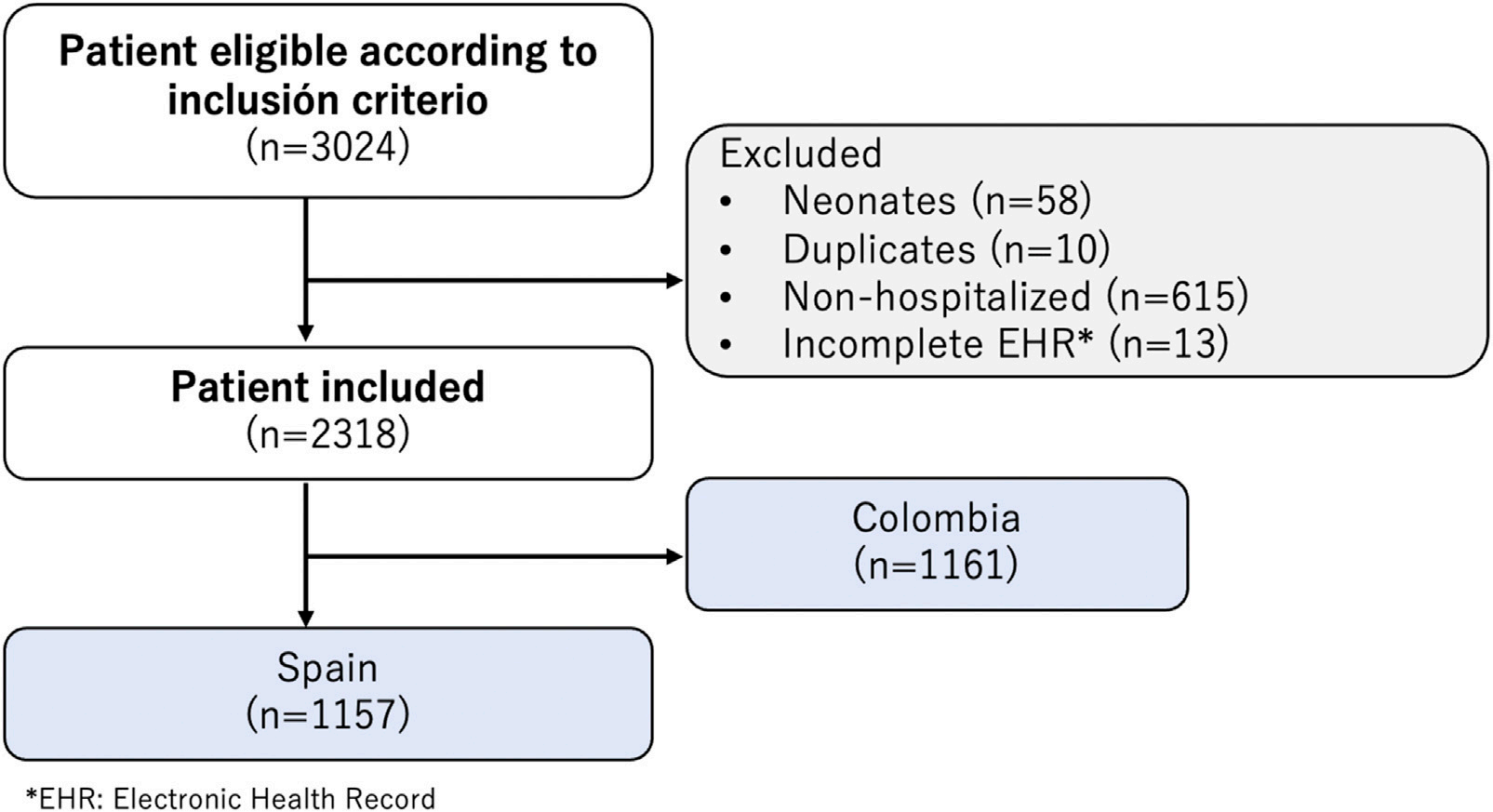
Flowchart of Epidemiological Study of COVID-19 in Children of the Spanish Society of Pediatrics (EPICO) cohort study and Institutions included (EPICO Study. Bogotá, Colombia, 2021).

The EPICO (Epidemiological study of respiratory infections caused by SARS-CoV-2 infection in pediatric population) is a multicentric cohort study conducted to investigate the behavior and hospital spectrum of SARS-CoV-2 infection in the pediatric population, including its evolution, severity factors, and outcomes between April 2020 and November 2021. The cohort included 13 hospitals in Colombia and 55 hospitals in Spain. The Colombian hospitals were predominantly located in the capital, Bogotá D.C., while the Spanish hospitals were spread across 13 provinces. Figure 1 shows the flowchart of EPICO cohort, and Supplementary Material S1 includes additional information about the institutions included. More information about the study, can be found in [17].

### Participants

Clinical records from eligible patients (children aged 29 days to 17 years, hospitalized with confirmed SARS-CoV-2 infection by either method [PCR (Protein Chain Reaction), antigen detection, or antibody testing Immunoglobulin G (IgG)/Immunoglobulin M (IgM +)] were included. The participants were admitted to the emergency room, inpatient ward, or pediatric critical care unit (PICU). Clinical records that met the inclusion criteria were selected by clinical research staff through daily review. Patients who were admitted to the emergency room and did not require hospital admission were excluded. We did not perform a sample size calculation because we had a significant amount of uncertainty regarding the epidemiology of the disease. The number of pediatric patients hospitalized in participating hospitals in Colombia and Spain with confirmed SARS-CoV-2 infection determined the sample size for this study. The patients were followed throughout their hospitalization, with no outpatient monitoring conducted.

### Data Sources

The data corresponding to the variables selected for the study were recorded retrospectively from clinical records and standardized in a digital format (REDCap), which only the researchers had access to maintain confidentiality. The development of COVID-19 infection was analyzed as the primary outcome, and five main categories were considered for the collection of variables: demographic characteristics, comorbidities, signs and symptoms upon admission, laboratory variables, and variables related to patients’ evolution and outcomes (treatment, medication, complications, hospital stay, and ventilation).

### Variables and Categorizing Variables

A stepwise protocol was developed to select and organize variables for cluster analysis. All variables were collected from primary sources within the participating institutions.

A total of 255 variables were collected and classified into three main categories according to the literature review: demographic, clinical, and complementary variables. Subsequently, the research group selected 25 variables through clinical experience and an individual literature review, followed by a meeting and discussion to ensure no variables of potential clinical value were overlooked when classifying the sample. The three factors considered for filtering the variables were:

- Clinical manifestations, management, and outcomes described and observed for COVID-19 and other acute respiratory diseases based on age.
- Severity of acute disease according to microbiological codetection and characteristics of the microorganisms involved, as seen in other diseases such as acute bronchitis [18–20].
- Correlation between the severity of the disease and the patient’s outcomes with their comorbidities, a finding that is notable in COVID-19 among adults [4, 21–23].

### Data Analysis

We used descriptive statistics for all variables of interest. For qualitative variables (all variables except length of hospitalization), we used absolute and relative frequencies. Quantitative variables were expressed by measures of central tendency and dispersion (appropriate according to their distribution).

To classify the different clinical phenotypes of the presentation of COVID-19 in pediatric patients, a cluster analysis was performed based on an agglomerative hierarchical ward analysis. This technique is a type of hierarchical clustering method that builds nested clusters by repeatedly merging pairs of clusters that minimize the increase in the overall within-cluster variance. Specifically, Ward’s method focuses on minimizing the sum of squared differences within clusters, making it particularly effective for creating clusters with relatively similar sizes [24]. In this analysis, categorical and continuous variables can be used, but in our study, we focused exclusively on qualitative variables to assess the clinical phenotypes. The Gower coefficient, which is well-suited for mixed data types, was used to calculate the similarity between patients. This method does not require large sample sizes, but the number of clusters can be influenced by the number of variables and the underlying distribution of the data. Ward’s method is advantageous over other clustering methods due to its ability to minimized within-cluster variance; however, it can be sensitive to outliers and may requiere careful interpretation to avoid overfitting.

The optical number of clusters was initially determined by visually inspecting the dendrogram, a tree-like diagram that represents the order in which clusters are merged. This was further confirmed by using the Elbow method, which assesses the percentage of variance explaned as a function of the number of clusters, and the Silhouette coefficient, which measures how similar each observation is to its own cluster compared to other clusters [25]. To perform the comparative analysis between the clusters and the outcomes of interest, we used ANOVA, X^2^, or Fisher’s exact tests, Student’s t-test, or the Mann-Whitney test, depending on the data type and distribution, with a significance level set at 5%.

All statistical analyzes were performed in R (version 4.0.4). The Colombian and Spanish cohort databases were combined by including only patients hospitalized between April 2020 and November 2021. EPICO Spain and EPICO Colombia used the same collection instrument to ensure comparability between the two cohorts.

For more detailed information on the agglomerative hierarchical ward’s method, please refer to key publications such as Murtagh and Legendre [26] on hierarchical clustering algorithms, and Milligan [27] for a detailed comparison of Ward´s method with other clustering techniques.

## Results

A total of 2,318 patients were included in the study, with equal distribution from the Colombian and Spanish institutions (50% vs. 50% respectively, 55% were male). The age distribution showed that infants accounted for the highest proportion (36%), followed by adolescents (23%), preschoolers (21%) and school-aged children (20%). Most patients (71%) did not report any comorbidity, while 18% had a non-respiratory comorbidity, and the remaining patients reported any type of respiratory comorbidity. During diagnosis fever was the main symptom (71%), followed by cough (48%), rhinorrhea (45%), vomiting and nausea (26%), abdominal pain (21%), as the most common findings. As for treatment, 655 (27%) patients required steroid therapy, while 862 (36%) completed antibiotic treatment. Additional details are provided in Table 1.

**TABLE 1:** General characteristics of Epidemiological Study of COVID-19 in Children of the Spanish Society of Pediatrics (EPICO) cohort (EPICO Study. Bogotá, Colombia, 2021).

Variable	N = 23,181
Country	
Colombia	1,161 (50%)
Spain	1,157 (50%)
Age group	
Infant	826 (36%)
Preschooler	497 (21%)
School-aged children	470 (20%)
Adolescents	525 (23%)
Sex	
Male	1,268 (55%)
Female	1,049 (45%)
Non specified	1 (<0.1%)
Codetection	313 (14%)
Comorbidities	
None	1,484 (64%)
Non-respiratory comorbidity	490 (21%)
Respiratory comorbidity	344 (15%)
Treatment	
Steroid treatment	730 (31%)
Antiobiotic treatment	985 (42%)
Symptoms	
Fever	1,681 (73%)
Cough	1,130 (49%)
Rhinorrhea	1,016 (44%)
Wheezing	373 (16%)
Altered consciousness/confusion	120 (5.2%)
Abdominal pain	556 (24%)
Vomiting/Nausea	650 (28%)
Diarrhea	543 (23%)
Pale skin	133 (5.7%)
Acne	212 (9.1%)
Lymphadenopathy	77 (3.3%)
Capillary refil time > 2 s	79 (3.4%)
Shock	140 (6.0%)
1 n (%)	

**FIGURE 2:**
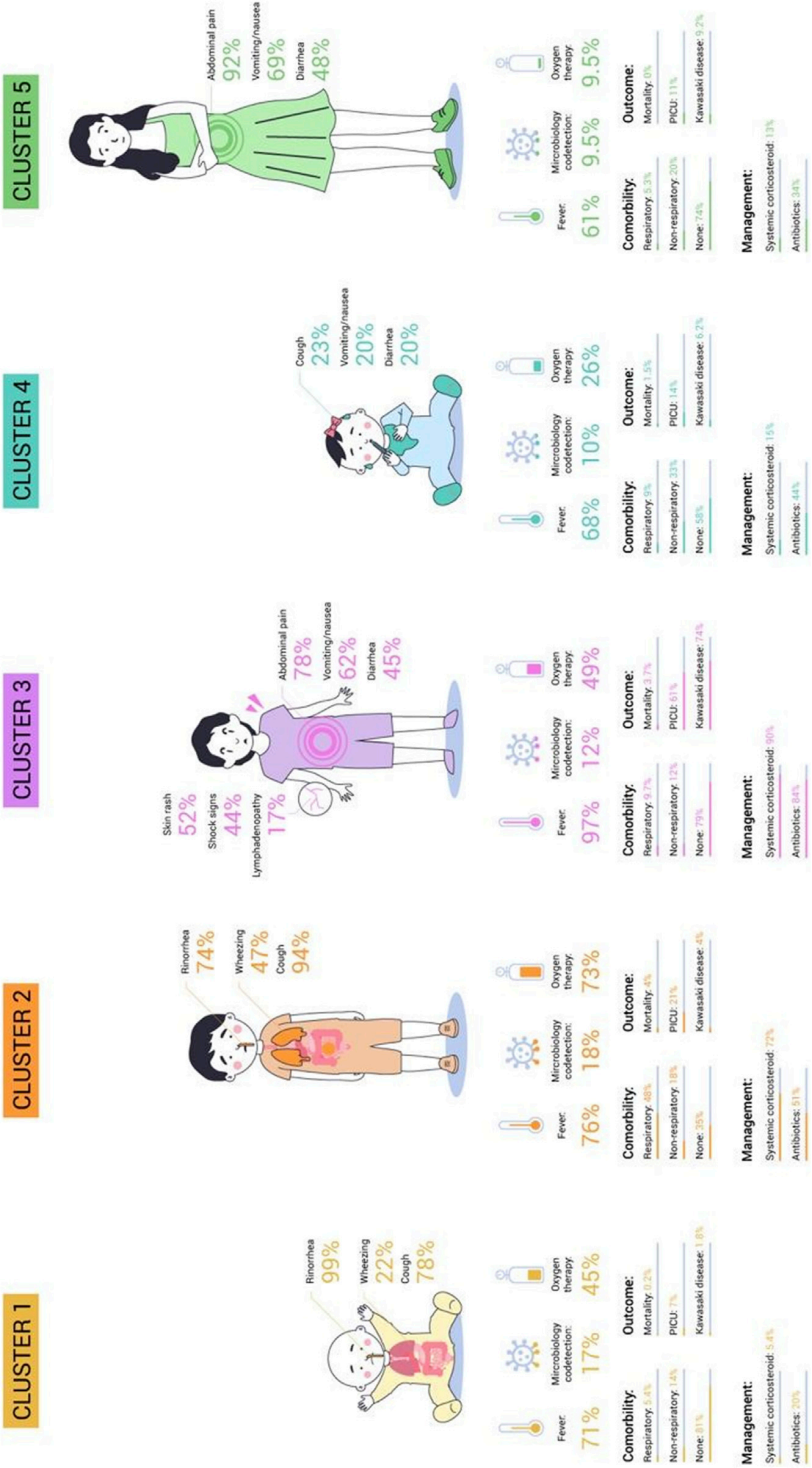
Clinical Phenotypes in Epidemiological Study of COVID-19 in Children of the Spanish Society of Pediatrics (EPICO) Cohort study (EPICO Study. Bogotá, Colombia, 2021).

The cluster analysis resulted in the identification of five distinct phenotype clusters (Figure 2). The determination of the number of clusters was based on the results from the dendrogram (Supplementary Material S4), where the cut-off point was chosen after observing the largest jump in dissimilarity, indicating the optimal number of clusters. This was further validated using the Elbow method, which showed a clear inflection point at five clusters. The Silhouette coefficient confirmed that this model provided the best balance between cohesion and separation among clusters, with a coefficient value indicating good overall clustering quality. Several potential models were initially identified; however, the selected five-cluster model was chosen for its clinical relevance and interpretability. This model effectively captured the diversity in clinical phenotypes while maintaining statistical robustness.

All clinical-epidemiologic characteristics were found to significantly differ between the groups (p-value <0.005). Cluster 4 was the most common (n = 744, 32%), while cluster 5 was the least common (n = 262, 11.3%). The specifics of each cluster are described individually below:

Cluster 1 (26.57%) predominantly consisted of infants without comorbidities. The primary symptoms observed in Cluster 1 were rhinorrhea (99%) and cough (78%), which are characteristic of upper respiratory tract symptoms, without wheezing. Patients in this cluster were treated mostly with oxygen therapy (45%) and had the lowest rates of admission to the PICU (7%) and mortality (0.2%).

Cluster 2 (18.5%) showed no significant age differences and was mostly male patients with respiratory comorbidities. The primary symptoms observed were cough (94%) and wheezing (47%), indicating a predominance of lower respiratory tract symptoms. Seventy-two percent of patients required systemic corticosteroids, 51% received antibiotic treatment. This cluster had the highest microbiological codetection rates (18%), need for oxygen therapy (73%), and morality rates (4%).

Cluster 3 (11.51%) was frequent in male patients without comorbidities. The most common symptoms were fever (97%) and gastrointestinal symptoms including abdominal pain (78%), vomiting/nausea (62%) and diarrhea (45%). Cutaneous rash (52%), shock signs (44%) and lymphadenopathy (17%) were also observed. This cluster had the greatest use of antibiotics (84%), systemic corticosteroids (90%) as well as the highest admission rate to the PICU (61%) and complications related to suspected Kawasaki like diseases (74%). The mortality rate in this cluster was 3.7%.

Cluster 4 (32.09%) showed no significant differences in age or sex and was characterized by patients with non-respiratory comorbidities. It was also characterized by fever (68%) and mild and non-specific symptoms related to the digestive and respiratory systems. This cluster also received antibiotics (44%) and the mortality rate was low (1.5%).

Cluster 5 (11.3%) consisted primarily of adolescents (39%) and female patients (58%) without comorbidities. Gastrointestinal symptoms including abdominal pain (92%), vomiting/nausea (69%) and diarrhea (48%) were prominent in this cluster. This cluster had the lowest rate of microbiology co-detection (9.5%) and received antibiotics in 34% of cases. The admission to a pediatric intensive care unit (PICU) and hospitalization rates were the lowest (11% and 3.7%, respectively). This cohort had no mortality.

In both countries, the five cluster groups were generally comparable, with few distinctions between them. Clusters 1 and 2 consisted primarily of Colombian patients (61% and 59% respectively), while cluster 3 was predominantly composed of Spanish patients (75%). Notably, there were disparities in mortality rates, with cluster 2 in Spain and cluster 3 in Colombia having the greatest mortality rates (Table 2).

**TABLE 2:** Total Phenotypes of COVID-19 presentation in hospitalized pediatric patients in Colombia and Spain (EPICO Study. Bogotá, Colombia, 2021).

Variable	N	1, N = 616^a^	2, N = 429^a^	3, N = 267^a^	4, N = 744^a^	5, N = 262^a^	p-value^b^
Country	2.318						<0.001
Colombia		374 (61%)	254 (59%)	66 (25%)	339 (46%)	128 (49%)	
Spain		242 (39%)	175 (41%)	201 (75%)	405 (54%)	134 (51%)	
Age group	2.318						<0.001
Infant		402 (65%)	112 (26%)	18 (6.7%)	266 (36%)	28 (11%)	
Preschooler		108 (18%)	141 (33%)	46 (17%)	157 (21%)	45 (17%)	
School aged		44 (7.1%)	96 (22%)	113 (42%)	129 (17%)	88 (34%)	
Adolescent		62 (10%)	80 (19%)	90 (34%)	192 (26%)	101 (39%)	
Sex	2.318						
Male		318 (52%)	277 (65%)	180 (67%)	383 (51%)	110 (42%)	
Female		298 (48%)	152 (35%)	87 (33%)	360 (48%)	152 (58%)	
Not specified		0 (0%)	0 (0%)	0 (0%)	1 (0.1%)	0 (0%)	
Codetection	2.318	103 (17%)	76 (18%)	31 (12%)	78 (10%)	25 (9.5%)	<0.001
Comorbidity status	2.318						<0.001
No comorbidities		496 (81%)	149 (35%)	210 (79%)	434 (58%)	195 (74%)	
Non respiratory comorbidity		87 (14%)	76 (18%)	31 (12%)	243 (33%)	53 (20%)	
Respiratory comorbidity		33 (5.4%)	204 (48%)	26 (9.7%)	67 (9.0%)	14 (5.3%)	
Treatment							
Systemic corticosteroids	2.318	33 (5.4%)	309 (72%)	241 (90%)	113 (15%)	34 (13%)	<0.001
Antibiotic	2.318	126 (20%)	219 (51%)	225 (84%)	327 (44%)	88 (34%)	<0.001
Symptoms							
History of fever	2.318	438 (71%)	324 (76%)	255 (96%)	505 (68%)	159 (61%)	<0.001
Cough	2.318	480 (78%)	404 (94%)	49 (18%)	173 (23%)	24 (9.2%)	<0.001
Rhinorrhea	2.318	607 (99%)	317 (74%)	33 (12%)	39 (5.2%)	20 (7.6%)	<0.001
Wheezing	2.318	138 (22%)	203 (47%)	9 (3.4%)	22 (3.0%)	1 (0.4%)	<0.001
Altered consciousness/confusion	2.318	17 (2.8%)	18 (4.2%)	44 (16%)	37 (5.0%)	4 (1.5%)	<0.001
Abdominal pain	2.318	47 (7.6%)	20 (4.7%)	208 (78%)	41 (5.5%)	240 (92%)	<0.001
Vomiting/Nausea	2.318	101 (16%)	52 (12%)	166 (62%)	149 (20%)	182 (69%)	<0.001
Diarrhoea	2.318	115 (19%)	36 (8.4%)	120 (45%)	146 (20%)	126 (48%)	<0.001
Pale/mottled skin	2.318	25 (4.1%)	16 (3.7%)	53 (20%)	33 (4.4%)	6 (2.3%)	<0.001
Skin rash	2.318	9 (1.5%)	5 (1.2%)	138 (52%)	56 (7.5%)	4 (1.5%)	<0.001
Lymphadenopathy	2.318	4 (0.6%)	3 (0.7%)	46 (17%)	23 (3.1%)	1 (0.4%)	<0.001
Capillary refill time >2 s?	2.318	4 (0.6%)	7 (1.6%)	59 (22%)	5 (0.7%)	4 (1.5%)	<0.001
Shock signs	2.318	3 (0.5%)	6 (1.4%)	118 (44%)	12 (1.6%)	1 (0.4%)	<0.001
Clinical outcomes							
Hospital time in days, median (IQR)	2.287	4 (3.6)	5 (4.9)	9 (6.12)	5 (3.8)	4 (3.7)	<0.001
Admission to PICU (yes)^c^	2.318	43 (7.0%)	91 (21%)	163 (61%)	107 (14%)	28 (11%)	<0.001
Oxygen therapy^c^	2.318	276 (45%)	312 (73%)	131 (49%)	196 (26%)	25 (9.5%)	<0.001
Kawasaki complication	2.318	11 (1.8%)	17 (4.0%)	197 (74%)	46 (6.2%)	24 (9.2%)	<0.001
Death	2.318	1 (0.2%)	17 (4.0%)	10 (3.7%)	11 (1.5%)	0 (0%)	<0.001

^a^ n (%); Median (IQR).

^b^ Pearson’s Chi-squared tests; Kruskal-Wallis rank sum test; Fisher’s exact test.

^c^ Missing values categorized as “No;” Pearson’s Chi-squared test; Kruskal-Wallis rank sum test; Fisher’s exact test.

In terms of clinical characteristics, the predominance of cough, fever, shortness of breath, nasal discharge and wheezing as the most frequent symptoms observed among the patients. For more details on each of the clusters by country please refer to the Supplementary Material S2, S3.

## Discussion

This study presents the findings of the only large-scale study conducted in Spanish-speaking hospitals, providing insights into the phenotypes of COVID-19 disease in infants and adolescents. Five distinct clinical phenotypes of COVID-19 disease were identified. Cluster 1 (26.57%) consisted of infants without comorbidities, with low PICU admission and mortality rates. Cluster 2 (18.5%) had respiratory comorbidities, high co-detection, and mortality rates. Cluster 3 (11.51%) showed fever, gastrointestinal symptoms, and high PICU admission. Cluster 4 (32.09%) had mild unspecific symptoms and low mortality. Cluster 5 (11.3%) included adolescents without comorbidities, with low co-detection and hospitalization rates. Comparable findings were observed in both countries.

Swann et al. described three clusters of clinical phenotypes: discrete respiratory illness, systemic mucocutaneous enteric illness, and neurological clusters [8]. Similarly, Cobos-Carrascosa et al. identified three phenotypes: lower respiratory, gastrointestinal, and flu-like, based on the EPICO-AE cohort [2]. However, it is important to note that these previous studies focused primarily on describing the presentation of the disease and did not provide a comprehensive description of variables such as treatment and outcomes. In contrast, our study not only categorized the different phenotypes but also incorporated treatment and outcome variables, which are crucial for guiding clinical management and prognosis.

In our study, cluster 1 was characterized by moderate upper respiratory symptoms correlating with previously published data on COVID-19 in the pediatric population, indicating that most pediatric patients are asymptomatic or have mild respiratory disease [3, 28–30]. Cluster 2 was characterized by lower respiratory symptoms with fever, cough, and wheezing as predominant symptoms, which is consistent with the reported in literature (26. The increased incidence of respiratory comorbidities and higher mortality rates can explain the more severe presentation of respiratory disease in Cluster 2 compared to Cluster 1. This is consistent with the findings of respiratory comorbidities, cardiac, cardiorespiratory and gastrointestinal complications as significant risk factors for the disease´s severe complications and admission to the Pediatric Intensive Care Unit (PICU) [31, 32].

Most patients in Cluster 3 display clinical characteristics consistent with MIS-C (Multisystem Inflammatory Syndrome in Children), a severe form of COVID-19 presentation in response to an intensive inflammatory response. This syndrome has been thoroughly described in the medical literature [9] and has also been observed in infections caused by other viruses, such as adenovirus [33, 34].

In Cluster 4, fever was the primary symptom of the disease, along with other non-specific symptoms. This could be explained by the fact that individuals in this cohort were tested for SARS-CoV-2 as part of routine evaluations in which COVID-19 was not the primary diagnosis. Instead, SARS-CoV-2 infection was discovered by accident. Cluster 5 was characterized by gastrointestinal symptoms and included predominantly female adolescents. Diarrhea has been reported as a significant symptom in COVID-19 infected patients. According to a study by Poeta M. et al., the global prevalence of this symptom is 18.9%, with a higher prevalence in children aged 5–11 years [35].

Other studies comparing clinical characteristics in children with SARS-CoV-2 infection identified manifestations such as myalgia, headache or dysgeusia but generally with low sensitivity [36–38]. All the differences between these studies and ours could be explained by the inclusion of hospitalized patients.

One strength of the study is the sample size, which includes two pediatric populations with different healthcare systems, cultural and socio-demographic characteristics. Similarly, it was conducted during a significant period of the pandemic, encompassing 2 years of data collection that captured both peaks and valleys of the disease. Furthermore, information from all age groups is included.

On the other hand, this study has some limitations, predominantly attributable to the selection of patient cohort from institutions that participate. Therefore, the information was retrieved retrospectively from the patient’s medical records without any active data collection. In addition, the participating institutions lacked a standardized protocol for disease management.

The identification of distinct clinical phenotypes in our study offers significant potential to improve the management of pediatric COVID-19. Recognizing the various ways in which SARS-CoV-2 manifests in children enables more precise treatment strategies. For instance, understanding that infants without comorbidities generally have low PICU admission and mortality rates (Cluster 1) can help clinicians avoid unnecessary interventions. Conversely, identifying high-risk groups, such as those with respiratory comorbidities and higher mortality rates (Cluster 2), emphasizes the importance of vigilant monitoring and early intervention.

These findings provide a foundation for developing personalized clinical guidelines that enhance risk stratification and resource allocation. This could involve creating in the future a risk classification system tailored to the pediatric population with SARS-CoV-2, incorporating factors like age, comorbidities, and clinical presentation. Such an approach would improve personalized care and optimize hospital resource allocation, ultimately leading to better patient outcomes.

### Conclusion

Our study successfully identified five phenotypic patterns of SARS-CoV-2 infection in hospitalized pediatric population, encompassing clinical manifestations, management, and prognosis. This information could be utilized to implement differential approaches, classify risks, and define individualized management strategies for patients in this age group. Efforts should be focused on devising and implementing management guidelines, as well as preventing the spread of infectious diseases.

## Data Availability

The datasets used and/or analyzed during the current study are available from the corresponding author on reasonable request.
