# Corrigendum: Phenotypic Variation in Disease Severity Among Hospitalized Pediatric Patients With COVID-19: Assessing the Impact of COVID-19 in the EPICO Study

**DOI:** 10.3389/ijph.2025.1608555

**Published:** 2025-04-22

**Authors:** María Camila Sossa-Alarcón, Mónica Paola Gutiérrez, Natalia Becerra, Luz Yessenia Ortegon, María Camila David, Melisa Naranjo Vanegas, Gabriela Friedrich, Pablo Vásquez-Hoyos, María Lucía Mesa-Rubio, Luis Miguel Navarro-Ramirez, Sergio Moreno-Lopez, Olga Lucía Baquero, Luz Marina Mejía, Juan Gabriel Piñeros, Sonia Restrepo-Gualteros, Carlos Álvarez-Moreno, Alejandro Díaz-Díaz, Iván Gutierrez-Tobar, Andrés Camilo Mesa, William Ricardo Bachiller Tuta, Clara Esperanza Galvis Diaz, Martha Africano, José Manuel Nieto, Paola Marsela Pérez Camacho, Claudia Beltrán-Arroyave, Rosalba Vivas Trochez, Irati Gastesi, Cinta Moraleda, Alfredo Tagarro García, Blanca Herrero, Lourdes Calleja, Carlos Grasa, Paula Rodriguez, Susana Melendo, Antoni Soriano-Arandes, Irene Gómez Pastrana, Sonsoles García García, Victoria Fumado, Andrea Ramírez Varela

**Affiliations:** ^1^ Pediatrics Department, Universidad de los Andes and Fundación Santa Fe de Bogotá, Bogotá, Colombia; ^2^ School of Medicine, Universidad de los Andes, Bogotá, Colombia; ^3^ Medical Imagine & AI group - Bioscience Center, Ayudas Diagnósticas Sura, Medellín, Colombia; ^4^ Red Colaborativa Pediátrica de Latinoamérica (LaRed Network), Montevideo, Uruguay; ^5^ School of Medicine, Universidad Nacional de Colombia, Bogotá, Colombia; ^6^ Cancer and Molecular Medicine Research Group (CAMMO), Bogotá, Colombia; ^7^ Department of Pediatrics, Clínica Infantil Colsubsidio, Bogotá, Colombia; ^8^ Department of Pediatrics, Instituto de Ortopedia Infantil Roosevelt, Bogotá, Colombia; ^9^ Clínica Universitaria Colombia, Clínica Colsanitas Grupo Keralty, Bogotá, Colombia; ^10^ Department of Pediatrics, Hospital Pablo Tobón Uribe, Medellín, Colombia; ^11^ Department of Pediatrics, Hospital General de Medellín, Medellín, Colombia; ^12^ Red Neumocolombia, Bogotá, Colombia; ^13^ Clínica Infantil Santa María del Lago, Clínica Colsanitas Grupo Keralty, Bogotá, Colombia; ^14^ Department of Pediatrics, Hospital Infantil San José, Bogotá, Colombia; ^15^ Department of Pediatrics, Fundación Universitaria de Ciencias de la Salud, Bogotá, Colombia; ^16^ Department of Pediatrics, Sociedad de Cirugía de Bogotá – Hospital de San José, Bogotá, Colombia; ^17^ Department of Pediatrics, Hospital Militar Central, Bogotá, Colombia; ^18^ Department of Pediatrics, Clínica Materno Infantil San Luis, Bucaramanga, Colombia; ^19^ Department of Pediatrics, Universidad Industrial de Santander, Bucaramanga, Colombia; ^20^ Department of Pediatrics, Hospital Regional de la Orinoquía, Yopal, Colombia; ^21^ Pediatric Infectious Diseases Department, Fundación Valle del Lili, Cali, Colombia; ^22^ Faculty of Health Sciences, Universidad Icesi, Cali, Colombia; ^23^ Department of Pediatrics, Clínica del Rosario, Medellín, Colombia; ^24^ School of Medicine, Universidad de Antioquia, Medellín, Colombia; ^25^ Department of Pediatrics, Clínica SOMA, Medellín, Colombia; ^26^ Fundación Investigación Biomédica, Hospital Universitario 12 de Octubre (FIBH120), Instituto de Investigación, Hospital 12 de Octubre (imas12), Madrid, Spain; ^27^ Department of Pediatrics, Infanta Sofía University Hospital, European University of Madrid, Madrid, Spain; ^28^ Department of Pediatrics, Hospital Universitario Niño Jesús, Madrid, Spain; ^29^ Department of Pediatrics, Hospital Universitario La Paz, Madrid, Spain; ^30^ Department of Pediatrics, Hospital Universitario Vall D´Hebron, Barcelona, Spain; ^31^ Department of Pediatrics, Hospital Universitario Sant Joan de Deu, Barcelona, Spain; ^32^ Department of Epidemiology, School of Public Health, The University of Texas Health Science Center at Houston, Houston, TX, United States; ^33^ Department of Pediatrics, McGovern Medical School, University of Texas Health Sciences Center at Houston (UTHealth), Houston, TX, United States; ^34^ School of Public Health, Center for Health Equity, University of Texas Health Science Center at Houston, Houston, TX, United States

**Keywords:** COVID-19, pediatrics, inpatients, cluster analysis, SARS-CoV-2 variants

In the published article, there was an error in **Affiliation 4**. Instead of “Red Colaborativa Pediátrica de Latinoamérica (LaRed Network), Universidad Nacional de Colombia, Bogotá, Colombia” it should be “Red Colaborativa Pediátrica de Latinoamérica (LaRed Network), Montevideo, Uruguay.”

In the published article, there was an error in **Affiliation 8**. Instead of “Department of Pediatrics, Instituto Roosevelt, Bogotá, Colombia” it should be “Department of Pediatrics, Instituto de Ortopedia Infantil Roosevelt. Bogotá, Colombia.”

In the published article, there was an error in **Affiliation 15**. Instead of “Fundación Universitaria de Ciencias de la Salud, Sociedad de Cirugía de Bogotá – Hospital de San José, Bogotá, Colombia” it should be “Department of Pediatrics, Fundación Universitaria de Ciencias de la Salud, Bogotá, Colombia.”

In the published article, there was an error in **Affiliation 18**. Instead of “Clínica Materno Infantil San Luis, Universidad Industrial de Santander, Bucaramanga, Colombia” it should be “Department of Pediatrics, Clínica Materno Infantil San Luis, Bucaramanga, Colombia.”

In the published article, there was an error in **Affiliation 21**. Instead of “Department of Pediatrics, Fundación Valle de Lili, Cali, Colombia” it should be “Pediatric Infectious Diseases Department, Fundación Valle del Lili, Cali, Colombia.”

In the published article, there was an error in **Affiliation 23**. Instead of “Clínica del Rosario, Universidad de Antioquia, Medellín, Colombia” it should be “Department of Pediatrics, Clínica del Rosario. Medellín, Colombia.”

In the published article, there was an error in **Affiliation 25**. Instead of “Clínica SOMA, Medellín, Colombia” it should be “Department of Pediatrics, Clínica SOMA, Medellín, Colombia.”

In the published article, there was an error in **Affiliation 32**. Instead of “Department of Epidemiology and Department of Pediatrics, School of Public Health, Center for Health Equity, University of Texas Health Science Center at Houston, Houston, TX, United States” it should be “Department of Epidemiology, School of Public Health, The University of Texas Health Science Center at Houston, Houston, Texas, USA.”

In the published article, there was an error regarding the affiliations for:

Melisa Naranjo Vanegas: As well as having the affiliation “School of Medicine, Universidad de los Andes. Bogotá, Colombia”, she should also have the affiliation “Medical Imagine & AI group - Bioscience Center, Ayudas Diagnósticas Sura. Medellín, Colombia”, instead of the affiliations “Red Colaborativa Pediátrica de Latinoamérica (LaRed Network), Universidad Nacional de Colombia, Bogotá, Colombia”, ^“^Department of Pediatrics, Clinica Infantil Colsubsidio, Bogotá, Colombia”, “Department of Pediatrics, Instituto Roosevelt, Bogotá, Colombia”, ^“^Department of Pediatrics, Hospital Pablo Tobón Uribe–Hospital General de Medellín, Medellín, Colombia”, “School of Medicine, Universidad Nacional and Clínica Universitaria Colombia, Bogotá, Colombia”, “Department of Pediatrics, Clinica Colsanitas, Bogotá, Colombia”, “Department of Pediatrics, Hospital Infantil San José, Bogotá, Colombia”, “Fundación Universitaria de Ciencias de la Salud, Sociedad de Cirugía de Bogotá – Hospital de San José, Bogotá, Colombia”, “Department of Pediatrics, Hospital Militar Central, Bogotá, Colombia”, “Clínica Materno Infantil San Luis, Universidad Industrial de Santander, Bucaramanga, Colombia”, “Department of Pediatrics, Hospital Regional de la Orinoquia, Yopal, Colombia”, “Department of Pediatrics, Fundación Valle de Lili, Cali, Colombia”, “Clínica del Rosario, Universidad de Antioquia, Medellín, Colombia”, “Clínica SOMA, Medellín, Colombia”, “Fundación Investigación Biomédica, Hospital Universitario 12 de Octubre (FIBH120), Instituto de Investigación, Hospital 12 de Octubre (imas12), Madrid, Spain”, “Department of Pediatrics, Infanta Sofía University Hospital, European University of Madrid, Madrid, Spain”, “Department of Pediatrics, Hospital Universitario Niño Jesús, Madrid, Spain”, “Department of Pediatrics, Hospital Universitario La Paz, Madrid, Spain”, “Department of Pediatrics, Hospital Universitario Vall D´Hebron, Barcelona, Spain”, “Department of Pediatrics, Hospital Universitario Sant Joan de Deu, Barcelona, Spain”, “Department of Epidemiology and Department of Pediatrics, School of Public Health, Center for Health Equity, University of Texas Health Science Center at Houston, Houston, TX, United States” and “Bioscience Center, Ayudas Diagnósticas Sura, Medellín, Colombia.”

Pablo Vásquez-Hoyos: As well as having the affiliation “School of Medicine, Universidad Nacional de Colombia, Bogotá, Colombia”, he should also have the affiliation “Red Colaborativa Pediátrica de Latinoamérica (LaRed Network), Montevideo, Uruguay.”

Luis Miguel Navarro-Ramirez: As well as having the affiliation “School of Medicine, Universidad Nacional de Colombia, Bogotá, Colombia”, he should also have the affiliation “Cancer and Molecular Medicine Research Group (CAMMO), Bogotá, Colombia” instead of the affiliations “Red Colaborativa Pediátrica de Latinoamérica (LaRed Network), Universidad Nacional de Colombia, Bogotá, Colombia”, ^“^Department of Pediatrics, Clinica Infantil Colsubsidio, Bogotá, Colombia”, “Department of Pediatrics, Instituto Roosevelt, Bogotá, Colombia”, ^“^Department of Pediatrics, Hospital Pablo Tobón Uribe–Hospital General de Medellín, Medellín, Colombia”, “School of Medicine, Universidad Nacional and Clínica Universitaria Colombia, Bogotá, Colombia”, “Department of Pediatrics, Clinica Colsanitas, Bogotá, Colombia”, “Department of Pediatrics, Hospital Infantil San José, Bogotá, Colombia”, “Fundación Universitaria de Ciencias de la Salud, Sociedad de Cirugía de Bogotá–Hospital de San José, Bogotá, Colombia”, “Department of Pediatrics, Hospital Militar Central, Bogotá, Colombia”, “Clínica Materno Infantil San Luis, Universidad Industrial de Santander, Bucaramanga, Colombia”, “Department of Pediatrics, Hospital Regional de la Orinoquia, Yopal, Colombia”, “Department of Pediatrics, Fundación Valle de Lili, Cali, Colombia”, “Clínica del Rosario, Universidad de Antioquia, Medellín, Colombia”, “Clínica SOMA, Medellín, Colombia”, “Fundación Investigación Biomédica, Hospital Universitario 12 de Octubre (FIBH120), Instituto de Investigación, Hospital 12 de Octubre (imas12), Madrid, Spain”, “Department of Pediatrics, Infanta Sofía University Hospital, European University of Madrid, Madrid, Spain”, “Department of Pediatrics, Hospital Universitario Niño Jesús, Madrid, Spain”, “Department of Pediatrics, Hospital Universitario La Paz, Madrid, Spain”, “Department of Pediatrics, Hospital Universitario Vall D´Hebron, Barcelona, Spain”, “Department of Pediatrics, Hospital Universitario Sant Joan de Deu, Barcelona, Spain”, “Department of Epidemiology and Department of Pediatrics, School of Public Health, Center for Health Equity, University of Texas Health Science Center at Houston, Houston, TX, United States”, “Bioscience Center, Ayudas Diagnósticas Sura, Medellín, Colombia” and “Cancer and Molecular Medicine Research Group (CAMMO), Bogotá, Colombia.”

Carlos Álvarez-Moreno: As well as having the affiliation “School of Medicine, Universidad Nacional de Colombia, Bogotá, Colombia”, he should also have the affiliation “Clínica Universitaria Colombia, Clínica Colsanitas Grupo Keralty, Bogotá, Colombia” instead of the affiliation “Department of Pediatrics, Hospital Pablo Tobón Uribe – Hospital General de Medellín, Medellín, Colombia.”

Alejandro Díaz-Díaz^:^ Instead of having the affiliation “School of Medicine, Universidad Nacional and Clínica Universitaria Colombia, Bogotá, Colombia” he should the affiliations “Department of Pediatrics, Hospital Pablo Tobón Uribe. Medellín, Colombia” and “Department of Pediatrics, Hospital General de Medellín. Medellín, Colombia.”

Iván Gutierrez-Tobar: Instead of having the affiliation “Department of Pediatrics, Clinica Colsanitas, Bogotá, Colombia”, he should have the affiliations “Department of Pediatrics, Clínica Infantil Colsubsidio. Bogotá, Colombia”, “Red Neumocolombia, Bogota, Colombia” and “Clínica Infantil Santa María del Lago, Clínica Colsanitas Grupo Keralty, Bogotá, Colombia.”

William Ricardo Bachiller Tuta: As well as having the affiliation “Department of Pediatrics, Fundación Universitaria de Ciencias de la Salud, Bogotá, Colombia”, he should also have the affiliation “Department of Pediatrics, Sociedad de Cirugía de Bogotá – Hospital de San José, Bogotá, Colombia.”

Martha Africano: As well as having the affiliation “Department of Pediatrics, Clínica Materno Infantil San Luis. Bucaramanga, Colombia” she should also have the affiliation “Department of Pediatrics, Universidad Industrial de Santander, Bucaramanga, Colombia.”

Paola Marsela Pérez Camacho: As well as having the affiliation “Pediatric Infectious Diseases Department, Fundación Valle de Lili. Cali, Colombia”, she should also have the affiliation “Faculty of Health Sciences, Universidad Icesi. Cali, Colombia.”

Claudia Beltrán-Arroyave: As well as having the affiliation “Department of Pediatrics, Clínica del Rosario. Medellín, Colombia” she should also have the affiliation “School of Medicine, Universidad de Antioquia. Medellín, Colombia.”

Andrea Ramírez Varela: As well as having the affiliation “Department of Epidemiology, School of Public Health, The University of Texas Health Science Center at Houston, Houston, Texas, USA” she should also have the affiliations “Department of Pediatrics, McGovern Medical School, University of Texas Health Sciences Center at Houston (UTHealth), Houston, Texas, USA” and “School of Public Health, Center for Health Equity, University of Texas Health Science Center at Houston, Houston, Texas, USA.”

The authors apologize for these errors and state that this does not change the scientific conclusions of the article in any way. The original article has been updated.

