# Supplementary material for: Phenotypic Variation in Disease Severity Among Hospitalized Pediatric Patients With COVID-19: Assessing the Impact of COVID-19 in the EPICO Study
Source: Int J Public Health. 2025 Mar 18;70:1607246. doi: 10.3389/ijph.2025.1607246 (PMC11959304; doi:10.3389/ijph.2025.1607246)

**SUPPLEMENTARY MATERIAL**

**Supplementary appendix 1.** List of Institutions involved in EPICO study.

| *EPICO-AEP* |
| --- |
| *Madrid, Parla, Alcalá de Henares, Majadahonda, Aranjuez, Alcorcon, Fuenlabrada, Fuenlabrada, Leganez, Arganda del Rey, Torrejón de Ardoz, Collado Villalba, Province of Madrid (28) – Community of Madrid* |
| **Hospital Universitario 12 de octubre**  **Hospital Universitario La Paz**  **Hospital Cínico San Carlos**  **Hospital General Gregorio Marañón**  **Hospital Universitario de Getafe**  **Hospital Universitario Infanta Sofía**  **Hospital Universitario Ramón y Caja**  **Hospital Universitario Fundación Jiménez Díaz**  **Hospital Universitario Niño Jesús**  **Hospital Infanta Cristina**  **Hospital Universitario Puerta de Hierro**  **Hospital Universitario Fundación Alcorcón**  **Hospital Universitario del Tajo**  **Hospital Universitario de Fuenlabrada**  **Hospital Universitario Infanta Elena**  **Hospital Universitario Infanta Leonor**  **Hospital Universitario Severo Ochoa**  **Hospital Universitario del Sureste**  **Hospital Universitario de Torrejón**  **Hospital General de Villalba**  **Hospital Universitario Rey Juan Carlos**  **Hospital Central de la Defensa Gómez Ulla**  **Universidad Europea de Madrid**  **Hospital Quirón Salud Madrid**  **Hospital HM de Madrid**  **Hospital Vithas Madrid Pardo de Aravaca**  **Hospital Universitario La Moraleja**  **Hospital Universitario Sanitas la Zarzuela** |
| *Sevilla, Málaga, Córdoba, Granada, Province of Sevilla (6) – Community of Andalucía* |
| **Hospital Universitario Virgen del Rocío**  **Hospital Universitario de Málaga**  **Instituto Hispalense de Pediatría**  **Hospital Puerta del Mar**  **Hospital Universitario Virgen de las Nieves**  **Hospital Universitario Torrecárdenas** |
| *Badalona, Tarragona, Province of Barcelona, Reus (7) – Community of Cataluña* |
| **Hospital Germans Trials i Pujol**  **Hospital Vall d´Hebron**  **Hospital Universitario Sant Joan de Reus**  **Hospital Universitario San Joan de Deú**  **Xarxa Assitencial Universitària de Manres**  **Hospital Universitari Arnau de Vilanova** |
| *Pamplona, Province of Navarra – Community of Navarra.* |
| **Complejo Hospitalario de Navarra** |
| *El Palmar, Province of Murcia – Community of Murcia* |
| **Hospital Universitari Son Espases** |
| *Logroño, Province of La Rioja – Community of La Rioja.* |
| **Hospital San Pedro** |
| *Palma de Mallorca, Mallorca Island – Community of Balearic Islands.* |
| **Hospital Universitari Son Espases**  **Hospital Universitario Materno Infantil de las Palmas** |
| *Albacete, Toledo, Province of Albacete – Community of Castilla-La Mancha.* |
| **Hospital General Universitario de Albacete**  **Complejo Hospitalario de Toledo** |
| *Alicante, València, Province of Valencia (4) – Community of Valenciana.* |
| **Hospital San Pedro**  **Consorcio Hospital General Universitario de Valencia**  **Hospital Universitario Clínico de Valencia**  **Hospital General Universitario de Alicante** |
| *Mérida, Province of Badajoz – Community of Extremadura.* |
| **Hospital de Mérida** |
| *Valladolid, Province of Valladollid – Community of Castilla y León.* |
| **Complejo Hospitalario de Toledo**  **Hospital Virgen de la Luz**  **Hospital Universitario de Salamanca** |
| *EPICO-COLOMBIA* |
| Bucaramanga, Santander |
| **Clínica Materno Infantil San Luis** |
| Yopal, Casanare |
| **Hospital Regional de la Orinoquia** |
| Bogotá, Cundinamarca |
| **Fundación Santa Fe de Bogotá**  **Clínica Infantil Colsubsidio**  **Instituto de Ortopedia Infantil Roosevelt**  **Clínica Infantil Santa María del Lago Colsanitas**  **Clínica Pediátrica Colsanitas**  **Sociedad de Cirugía Hospital de San José**  **Hospital Infantil Universitario de San José**  **Hospital Militar Central** |
| Cali, Valle del Cauca |
| **Fundación Valle de Lili** |
| Medellín, Antioquia |
| **Clínica del Rosario**  **Sociedad Médica Antioqueña – Clínica SOMA** |

**Supplementary appendix 2.** Total Phenotypes of COVID-19 presentation in hospitalized pediatric patients in Colombia.

| Variable | N | 1, N = 374*^1^* | 2, N = 254*^1^* | 3, N = 66*^1^* | 4, N = 339*^1^* | 5, N = 128*^1^* | p-value*^2^* |
| --- | --- | --- | --- | --- | --- | --- | --- |
| Age group | 1.161 |  |  |  |  |  | <0.001 |
| Infant |  | 249 (67%) | 67 (26%) | 11 (17%) | 119 (35%) | 16 (12%) |  |
| Preschool |  | 62 (17%) | 86 (34%) | 8 (12%) | 83 (24%) | 22 (17%) |  |
| School aged |  | 22 (5.9%) | 58 (23%) | 22 (33%) | 56 (17%) | 39 (30%) |  |
| Adolescent |  | 41 (11%) | 43 (17%) | 25 (38%) | 81 (24%) | 51 (40%) |  |
| Sex | 1.161 |  |  |  |  |  | 0,042 |
| Male |  | 196 (52%) | 164 (65%) | 40 (61%) | 180 (53%) | 55 (43%) |  |
| Female |  | 178 (48%) | 90 (35%) | 26 (39%) | 159 (47%) | 73 (57%) |  |
| Not specified |  | 0 (0%) | 0 (0%) | 0 (0%) | 0 (0%) | 0 (0%) |  |
| Codetection | 1.161 | 54 (14%) | 37 (15%) | 5 (10%) | 40 (12%) | 13 (8.9%) | 0,3 |
| Comorbidity status | 1.161 |  |  |  |  |  | <0.001 |
| No comorbidities |  | 319 (85%) | 97 (38%) | 49 (74%) | 217 (64%) | 98 (77%) |  |
| Non respiratory comorbidity |  | 34 (9.1%) | 34 (13%) | 10 (15%) | 101 (30%) | 21 (16%) |  |
| Respiratory comorbidity |  | 21 (5.6%) | 123 (48%) | 7 (11%) | 21 (6.2%) | 9 (7.0%) |  |
| Treatment |  |  |  |  |  |  |  |
| Systemic corticosteroids | 1.161 | 22 (5.9%) | 203 (80%) | 56 (85%) | 51 (15%) | 10 (7.8%) | <0.001 |
| Antibiotic | 1.161 | 61 (16%) | 101 (40%) | 45 (68%) | 141 (42%) | 39 (30%) | <0.001 |
| Symptoms |  |  |  |  |  |  |  |
| History of fever | 1.161 | 255 (68%) | 181 (71%) | 60 (91%) | 223 (66%) | 76 (59%) | <0.001 |
| Cough | 1.161 | 306 (82%) | 237 (93%) | 11 (17%) | 72 (21%) | 11 (8.6%) | <0.001 |
| Rhinorrhea | 1.161 | 370 (99%) | 195 (77%) | 12 (18%) | 19 (5.6%) | 13 (10%) | <0.001 |
| Wheezing | 1.161 | 87 (23%) | 121 (48%) | 3 (4.5%) | 8 (2.4%) | 1 (0.8%) | <0.001 |
| Altered consciousness / confusion | 1.161 | 12 (3.2%) | 12 (4.7%) | 17 (26%) | 15 (4.4%) | 1 (0.8%) | <0.001 |
| Abdominal pain | 1.161 | 30 (8.0%) | 9 (3.5%) | 35 (53%) | 19 (5.6%) | 116 (91%) | <0.001 |
| Vomiting / Nausea | 1.161 | 59 (16%) | 27 (11%) | 34 (52%) | 77 (23%) | 86 (67%) | <0.001 |
| Diarrhoea | 1.161 | 73 (20%) | 20 (7.9%) | 29 (44%) | 73 (22%) | 65 (51%) | <0.001 |
| Pale/mottled skin | 1.161 | 13 (3.5%) | 11 (4.3%) | 18 (27%) | 13 (3.8%) | 2 (1.6%) | <0.001 |
| Skin rash | 1.161 | 5 (1.3%) | 3 (1.2%) | 20 (30%) | 22 (6.5%) | 1 (0.8%) | <0.001 |
| Lymphadenopathy | 1.161 | 2 (0.5%) | 1 (0.4%) | 8 (12%) | 13 (3.8%) | 0 (0%) | <0.001 |
| Capillary refill time > 2 seconds ? | 1.161 | 3 (0.8%) | 7 (2.8%) | 23 (35%) | 2 (0.6%) | 3 (2.3%) | <0.001 |
| Shock signs | 1.161 | 1 (0.3%) | 4 (1.6%) | 31 (47%) | 5 (1.5%) | 0 (0%) | <0.001 |
| Clinical outcomes |  |  |  |  |  |  |  |
| Hospital time in days, median (IQR) | 1.161 | 4(3,6) | 5(4,8) | 9(5,13) | 5(3,9) | 4(3,7) | <0.001 |
| Admission to PICU (yes) | 1.161 | 28(7.5%) | 67(26%) | 49(74%) | 71(21%) | 14(11%) | <0.001 |
| Oxygen therapy | 1.161 | 221(59%) | 213(84%) | 44(67%) | 127(37%) | 18(14%) | <0.001 |
| Kawasaki complication | 1.161 | 7(1.9%) | 9(3.5%) | 29(44%) | 16(4.7%) | 2(1.6%) | <0.001 |
| Death | 1.161 | 1(0.3%) | 12(4.7%) | 7(11%) | 5(1.5%) | 0(0%) | <0.001 |
| *^1^* n (%); Median (IQR) | | | | | | | |
| *^2^* Pearson's Chi-squared tests; Fisher's exact test; Kruskal-Wallis rank sum test | | | | | | | |

**Supplementary appendix 3.** Total Phenotypes of COVID-19 presentation in hospitalized pediatric patients in Spain.

| Variable | N | **1**, N = 242*^1^* | **2**, N = 175*^1^* | **3**, N = 201*^1^* | **4**, N = 405*^1^* | **5**, N = 134*^1^* | **p-value***^2^* |
| --- | --- | --- | --- | --- | --- | --- | --- |
| Age group | 1.157 |  |  |  |  |  | <0.001 |
| **Infant** |  | 153 (63%) | 45 (26%) | 7 (3.5%) | 147 (36%) | 12 (9.0%) |  |
| **Preschool** |  | 46 (19%) | 55 (31%) | 38 (19%) | 74 (18%) | 23 (17%) |  |
| **School aged** |  | 22 (9.1%) | 38 (22%) | 91 (45%) | 73 (18%) | 49 (37%) |  |
| **Adolescent** |  | 21 (8.7%) | 37 (21%) | 65 (32%) | 111 (27%) | 50 (37%) |  |
| Sex | 1.157 |  |  |  |  |  |  |
| **Male** |  | 122 (50%) | 113 (65%) | 140 (70%) | 203 (50%) | 55 (41%) |  |
| **Female** |  | 120 (50%) | 62 (35%) | 61 (30%) | 201 (50%) | 79 (59%) |  |
| **Not specified** |  | 0 (0%) | 0 (0%) | 0 (0%) | 1 (0.2%) | 0 (0%) |  |
| Codetection | 1.157 | 48 (20%) | 39 (22%) | 25 (12%) | 39 (9.6%) | 13 (9.7%) | <0.001 |
| Comorbidity status | 1.157 |  |  |  |  |  | <0.001 |
| **No comorbidities** |  | 177 (73%) | 52 (30%) | 161 (80%) | 217 (54%) | 97 (72%) |  |
| **Non respiratory comorbidity** |  | 53 (22%) | 42 (24%) | 21 (10%) | 142 (35%) | 32 (24%) |  |
| **Respiratory comorbidity** |  | 12 (5.0%) | 81 (46%) | 19 (9.5%) | 46 (11%) | 5 (3.7%) |  |
| Treatment |  |  |  |  |  |  |  |
| **Systemic corticosteroids** | 1.157 | 11 (4.5%) | 106 (61%) | 185 (92%) | 62 (15%) | 24 (18%) | <0.001 |
| **Antibiotic** | 1.157 | 65 (27%) | 118 (67%) | 180 (90%) | 186 (46%) | 49 (37%) | <0.001 |
| Symptoms |  |  |  |  |  |  |  |
| **History of fever** | 1.157 | 183 (76%) | 143 (82%) | 195 (97%) | 282 (70%) | 83 (62%) | <0.001 |
| **Cough** | 1.157 | 174 (72%) | 167 (95%) | 38 (19%) | 101 (25%) | 13 (9.7%) | <0.001 |
| **Rhinorrhea** | 1.157 | 237 (98%) | 122 (70%) | 21 (10%) | 20 (4.9%) | 7 (5.2%) | <0.001 |
| **Wheezing** | 1.157 | 51 (21%) | 82 (47%) | 6 (3.0%) | 14 (3.5%) | 0 (0%) | <0.001 |
| **Altered consciousness / confusion** | 1.157 | 5 (2.1%) | 6 (3.4%) | 27 (13%) | 22 (5.4%) | 3 (2.2%) | <0.001 |
| **Abdominal pain** | 1.157 | 17 (7.0%) | 11 (6.3%) | 173 (86%) | 22 (5.4%) | 124 (93%) | <0.001 |
| **Vomiting / Nausea** | 1.157 | 42 (17%) | 25 (14%) | 132 (66%) | 72 (18%) | 96 (72%) | <0.001 |
| **Diarrhoea** | 1.157 | 42 (17%) | 16 (9.1%) | 91 (45%) | 73 (18%) | 61 (46%) | <0.001 |
| **Pale/mottled skin** | 1.157 | 12 (5.0%) | 5 (2.9%) | 35 (17%) | 20 (4.9%) | 4 (3.0%) | <0.001 |
| **Skin rash** | 1.157 | 4 (1.7%) | 2 (1.1%) | 118 (59%) | 34 (8.4%) | 3 (2.2%) | <0.001 |
| **Lymphadenopathy** | 1.157 | 2 (0.8%) | 2 (1.1%) | 38 (19%) | 10 (2.5%) | 1 (0.7%) | <0.001 |
| **Capillary refill time > 2 seconds ?** | 1.157 | 1 (0.4%) | 0 (0%) | 36 (18%) | 3 (0.7%) | 1 (0.7%) | <0.001 |
| **Shock signs** | 1.157 | 2 (0.8%) | 2 (1.1%) | 87 (43%) | 7 (1.7%) | 1 (0.7%) | <0.001 |
| Clinical outcomes |  |  |  |  |  |  |  |
| **Hospital time in day, median (IQR)** | 1.126 | 4(3,6) | 5(4,10) | 9(6,12) | 5(3,8) | 4(3,6) | <0.001 |
| **Admission to PICO (yes)*** | 1.157 | 15(6.2%) | 24(14%) | 114(57%) | 36(8.9%) | 14(10%) | <0.001 |
| **Oxygen therapy*** | 1.157 | 55(23%) | 99(57%) | 87(43%) | 69(17%) | 7(5.2%) | <0.001 |
| **Kawasaki complication** | 1.157 | 4(1.7%) | 8(4,6%) | 168(84%) | 30(7.4%) | 22(16%) | <0.001 |
| **Death** | 1.157 | 0(0%) | 5(2.9%) | 3(1.5%) | 6(1.5%) | 0(0%) | 0.032 |
| *^1^* n (%); Median (IQR) | | | | | | | |
| *^2^* Pearson's Chi-squared tests; Fisher's exact test; Kruskal-Wallis rank sum test | | | | | | | |

**Supplementary appendix 4.** Dendrogram


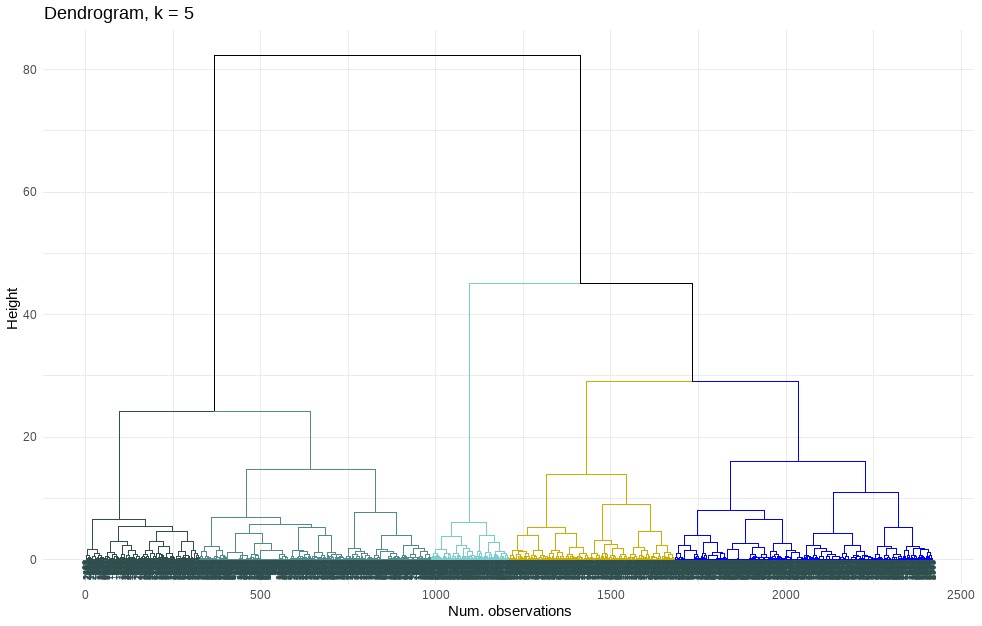

Supplement: Supplementary file 1 [file DataSheet1.docx]
